# Effect of Synthetic Low-Odor Thiol-Based Hardeners Containing Hydroxyl and Methyl Groups on the Curing Behavior, Thermal, and Mechanical Properties of Epoxy Resins

**DOI:** 10.3390/polym15132947

**Published:** 2023-07-04

**Authors:** Young-Hun Kim, Jeong Ju Baek, Ki Cheol Chang, Baek Soo Park, Won-Gun Koh, Gyojic Shin

**Affiliations:** 1 Green and Sustainable Materials R&D Department, Research Institute of Clean Manufacturing System, Korea Institute of Industrials Technology (KITECH), Yangdaegiro-gil 89, Ipjang-myeon, Cheonan-si 31056, Republic of Korea; kimyh@kitech.re.kr (Y.-H.K.);; 2 Department of Chemical and Biomolecular Engineering, Yonsei University, Yonsei-ro 50, Seodaemun-gu, Seoul 03722, Republic of Korea

**Keywords:** epoxy resin, thiol-based hardener, polysilsesquioxane backbone, air dilution olfaction method, low volatile organic compound (VOC)

## Abstract

A novel thiol-functionalized polysilsesqioxane containing hydroxyl and methyl groups was synthesized using a simple acid-catalyzed sol–gel method to develop an epoxy hardener with low odor, low volatile organic compound (VOC) emissions, and fast curing at low temperatures. The synthesized thiol-based hardeners were characterized using Fourier transform infrared spectroscopy, nuclear magnetic resonance, thermogravimetric analysis (TGA), and gel permeation chromatography and compared with commercially available hardeners in terms of odor intensity and VOC emissions using the air dilution olfaction method and VOC analysis. The curing behavior and thermal and mechanical properties of the epoxy compounds prepared with the synthesized thiol-based hardeners were also evaluated. The results showed that synthetic thiol-based hardeners containing methyl and hydroxyl groups initiated the curing reaction of epoxy compounds at 53 °C and 45 °C, respectively. In contrast, commercial thiol-based hardeners initiated the curing reaction at 67 °C. Additionally, epoxy compounds with methyl-containing synthetic thiol-based hardeners exhibited higher TGA at a 5% weight loss temperature (>50 °C) and lap shear strength (20%) than those of the epoxy compounds with commercial thiol-based hardeners.

## 1. Introduction

Epoxy resins are widely used thermosetting resins in various industries for various applications, such as coatings, adhesives, insulators, and matrixes for composite materials, because of their excellent electrical insulation, low molding shrinkage, good adhesion, and heat resistance [1,2,3,4,5]. However, their curing behaviors are complex and depend on various factors, such as temperature, molecular mobility, and crosslinking reactions [6,7,8]. Therefore, the study and prediction of their curing behavior are essential for the effective processing of thermosetting resins [9,10,11,12].

One-component epoxy adhesives, which are commonly used in electronics, require high temperatures (approximately 120 °C or higher) to cure. However, these high temperatures can cause thermal damage to optical components in electronics [13,14,15]. One strategy to solve this problem is lowering the curing temperature of epoxy resins. However, this can also prolong the curing time and reduce productivity. Therefore, low-temperature, fast-curing epoxy adhesives that can balance these factors must be developed [16,17,18].

Currently used hardeners for low-temperature fast-curing methods include cationic [19], acrylic [20], and thiol hardeners [21]. However, the main problem with cationic hardeners is that antimony-based cationic initiators used for curing are heavy metals subject to various environmental regulations. These heavy metals are difficult to handle and dispose of, and their use can negatively impact the environment [22,23]. In contrast, acrylic hardeners generally show fast-curing performance at temperatures above 120 °C [24]. However, their curing is unstable at low temperatures below 85 °C. This is because free radicals and oxygen can interfere with the curing process, causing the surface to not cure or reduce the surface strength [25]. Meanwhile, depending on the curing conditions, thiol-based hardeners are commonly used for the low-temperature fast curing of epoxy resins, which can be achieved within 30 min at 80–90 °C, within 10 min at over 90 °C, several min at 100 °C, or within tens of seconds to 1 min at 150 °C using different heat sources, such as a low-temperature oven, a hot plate, or an inline oven [26,27]. However, thiol-based hardeners produce a strong odor that reduces work comfort, and they take time to cure fully. Previous studies have reported the reduction of thiol odor through organic synthesis, but none have linked this to VOCs [28,29]. Therefore, in this paper, we not only quantified the reduction of thiol odor using olfactory measurements but also quantified the intensity of the odor of each compound using VOC measurements.

In present study, a novel thiol-functionalized polysilsesquioxane containing hydroxyl and methyl groups was synthesized to develop an epoxy hardener with low odor, low volatile organic compound (VOC) emission, and fast curing at low temperatures. The structures of the synthesized hardener were characterized using Fourier transform infrared spectroscopy (FTIR), nuclear magnetic resonance (NMR), thermogravimetric analysis (TGA), and gel permeation chromatography (GPC) and compared with those of commercial thiol hardeners in terms of odor intensity and VOC emissions. Dipentaerythritol hexakis (3-mercaptopropionate) (DPETMP) is a popular hardener for epoxy resins because of its low-temperature curing and low toxicity. It has 6 reactive thiol groups, which make it very efficient in forming cross-linked networks with epoxy groups. Therefore, DPETMP was selected as a control for this paper. Additionally, epoxy compounds were prepared after the addition of the thiol hardener, and their curing behavior and thermal and mechanical properties were evaluated. The experiments employed two main strategies: first, the optimal addition amount of the thiol-based hardener was determined; second, the aim was to evaluate the chemical characteristics of hydroxyl and methyl groups to select a more suitable thiol-based hardener. The thiol-based hardener with a methyl group showed a higher curing initiation temperature than that with the hydroxyl group in curing behavior. However, the conversion rate and curing time were superior for the hydroxyl group hardener. Meanwhile, the thermal stability and mechanical strength were superior for the hydroxyl-group-containing thiol hardener and existing commercially available thiol hardener. These results show that thiol-based hardeners, including methyl, are the best epoxy hardeners.

## 2. Experimental

### 2.1. Materials

Bisphenol A diglycidyl ether (DGBEA, YD-128) with an epoxy equivalent weight of 172–176 g/eq was obtained from Kukdo Chemical and dried under a vacuum at 80 °C for 3 h and stored in a desiccator before use. (3-Mercaptopropyl)trimethoxysilane [MPTMS, 96%], methoxytrimethylsilane [MTMS, 98%], 1-methylimidzole (1-MI), and dipentaerythritol hexakis (3-mercaptopropionate) [DPETMP, 93%] were purchased from (TCI Co. Inc, Chuo-ku, Tokyo, Japan.) All materials were used as received without further purification.

### 2.2. Synthesis of Thiol-Functionalized Polysilsesquioxane Containing a Hydroxyl Group (TFPH)

First, 6.19 g of hydrochloric acid (36.5% aqueous solution, 62 mmol) was added to the mixture of solvents (21.5 g of ethanol and 4.8 g of distilled water). Second, the mixture of monomers (3.73 g of (3-mercaptopropyl) trimethoxysilane [19 mmol]) was added to the mixture of solvents. Then, the solution was heated at 65 °C for 16 h under a nitrogen atmosphere. After the reaction, the reaction mixture was poured into distilled water several times for purification. Ethyl alcohol, H_2_O, methyl alcohol as by-products, and impurities were removed using a rotary evaporator, and the obtained product was dried in a vacuum oven at 100 °C overnight to obtain a transparent viscous liquid.

TFPH was a clear colorless liquid:

^1^H NMR (300 MHz, CDCl_3_, ppm): *δ* = 0.78 (m, Si–CH_2_), 1.25–1.42 (m, Si–CH_2_–CH_2_–CH_2_–SH), 1.66–1.76 (m, Si–CH_2_–CH_2_), 2.53–2.60 (t, Si–CH_2_–CH_2_–CH_2_), 3.77 (Si–OH). ^13^C NMR (100.62 MHz, CDCl_3_, ppm): *δ* = 10.24 (Si–CH_2_), 28.16 (Si–CH_2_–CH_2_–CH_2_–SH).

### 2.3. Synthesis of Thiol-Functionalized Polysilsesquioxane Containing a Methyl Group (TFPM)

**Scheme 1:**
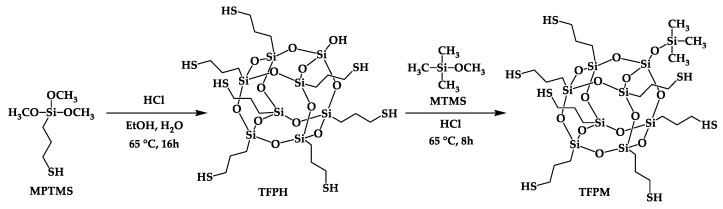
Synthesis schemes for TFPH and TFPM.

First, 6.19 g of hydrochloric acid (36.5% aqueous solution, 62 mmol) was added to the mixture of solvents (21.5 g of ethanol and 4.8 g of distilled water). Second, the mixture of monomers (3.73 g of (3-mercaptopropyl) trimethoxysilane [19 mmol]) was added to the solvent mixture. Then, the solution was heated at 65 °C for 16 h under a nitrogen atmosphere. After the reaction, methoxytrimethylsilane (0.38 g, 3.3 mmol) and 0.76 g of hydrochloric acid (36.5% aqueous solution, 8 mmol) were added, and the solution was stirred at 65 °C for another 8 h. After the reaction, the reaction mixture was poured into distilled water several times for purification. Ethyl alcohol, H_2_O, methyl alcohol as by-products, and impurities were removed using a rotary evaporator, and the obtained product was dried in a vacuum oven at 100 °C overnight to obtain a transparent viscous liquid. Scheme 1 shows the synthesis schemes for TFPH and TFPM.

TFPM was a clear colorless liquid:

^1^H NMR (300 MHz, CDCl_3_, ppm): *δ* = 0.06–0.19 (m, Si–CH_3_), 0.77–0.81 (s, Si–CH_2_), 1.66–1.76 (d, Si–CH_2_–CH_2_), 2.53–2.60 (d, Si–CH_2_–CH_2_–CH_2_), 1.25–1.42 (m, Si–CH_2_–CH_2_–CH_2_–SH). ^13^C NMR (100.62 MHz, CDCl_3_, ppm): *δ* = 1.39 (Si–CH_3_), 10.28 (Si–CH_2_), 28.25 (Si–CH_2_–CH_2_–CH_2_–SH).

### 2.4. Characterization of TFPH and TFPM

The structural analyses of TFPH and TFPM were performed using a Spectrum-400 FTIR spectrometer (Perkin Elmer, Waltham, MA, USA) with 100 scans in the wavenumber range of 650–4000 cm^−1^. The samples (10 μm) were dropped onto attenuated total reflectance crystals at room temperature. All spectra were adjusted by reducing CO_2_, removing noise, and fitting the baseline. ^1^H, ^13^C, and ^29^Si NMR spectra were obtained on a 300 MHz NMR instrument Avance 300 (Bruker, Billerica, MA, USA) at room temperature using CDCl_3_ as the solvent. 

The molecular weights of TFPH and TFPM were determined through size exclusion chromatography using an EcoSEC HLC-8320 GPC (TOSOH, Minato-ku, Tokyo, Japan). A sample solution of 0.15% (wt/vol) of TFPH or TFPM in tetrahydrofuran (THF) was injected into the GPC system. The separation was conducted on a combination of Guard Super MP (HZ)-M^+2^ and TSK gel Supermultipore HZ-M columns (150 mm × 4.6 mm, 3 μm). The mobile phase was high-performance-liquid-chromatography-grade THF flowing at a rate of 0.45 mL/min. The column temperature was set to 40 °C. The GPC system was calibrated using a series of thin polystyrene molecular weight standards with values of 580, 2980, 9960, 30,230, 69,650, 128,600, 325,600, and 660,500 Da.

The thermal stabilities of the samples were determined using a TGA-4000 thermal analyzer (Perkin Elmer, Waltham, MA, USA). Herein, 5–10 mg of samples were heated in a nitrogen environment at a rate of 10 °C/min from 30 °C to 800 °C.

The air dilution olfactory method was performed as follows: five filter papers (14 cm × 7 mm) were prepared, and three of them were immersed in the test solution for 5 min. The five filter papers were given to several healthy subjects, who were asked to select three of the filter papers that smelled the strongest. If four or more people correctly identified the three filter papers, they were considered preliminary judges. The filter papers used for the preliminary test were then discarded. Then, five people were selected to be odor-determination agents. The sample was diluted, and a sensory test was conducted with the odor-determination agents. The odor dilution factor was calculated as the geometric mean of the remaining values (three persons), excluding the maximum and minimum values. The sample was diluted with odorless air in steps of 10, 30, 100, 300, 1000, 3000, and so on, until the odor was no longer detectable. For example, in performing a 10-fold dilution, 300 mL of the sample was injected into a 3 L odorless air bag (syringe).

A thermal extractor TE (Gerstel, Linthicum Heights, MD, USA) with flow control (10–300 mL/min) was used to quantify the emission of total VOCs (TVOCs) from the samples. The VOCs were released by a carrier gas at a flow rate of 134 mL/min and were collected in adsorption tubes. Each 25 mg sample was placed in its own glass extraction tube. The TE consisted of an adjustable oven (room temperature) that heated the sample-containing glass tube (178 mm, diameter 13.6 mm). The “Methods for Measuring VOCs Emissions from Indoor and Building Materials–Solid Absorber Tubes and GC-MS/FID Method” ES 02603.1 were used for the VOC analysis. VOCs were purged under a steady flow of pure nitrogen gas on a Tenax TA adsorption tube (Supelco, Bellefonte, PA, USA) and micro pump MP-30 (SIBATA, Soka, Saitama, Japan). The thermal extraction procedure was conducted at 25 ± 5 °C for 30 min, and a total of 1 L of gas was sampled. Qualitative analysis was performed using individual calibration lines for the following compounds: TVOC, benzene, toluene, ethylbenzene, o-, m-, and p-xylene, and styrene. The TVOC concentration (µg/m^3^) was calculated using the toluene calibration curve for the total area of the chromatogram between *n*-hexane and *n*-hexadecane.

### 2.5. Preparation of Epoxy Compound

For preparing an epoxy composition, 10 g of epoxy resin and 10 phr of 1-methylimidazole were added to a mixing vessel. Then, three types of thiol-based hardeners (DPETMP, TFPH, and TFPM) were added at various weight percentages (wt%): 0, 10, 20, 30, and 40 wt%. The mixtures were mixed for 10 min using a Thinky mixer. After mixing, the mixtures were homogenized at 30 °C for 10 min using an IKA Ultra-Turrax T25 digital homogenizer (IKA, Baden-Württemberg, Freiburg, Germany) with a 10 mm dispersing tool operating at 20,000 rpm.

### 2.6. Characterization of the Epoxy Compound

A differential scanning calorimeter DSC-8500 (Perkin Elmer, Waltham, MA, USA) was used to investigate the curing behavior. A 5 to 10 g sample was placed in an aluminum pan for the DSC, and the experiment was conducted under a nitrogen atmosphere. The change in the heat of reaction at elevated and isothermal conditions was observed. The elevated temperature method was used to analyze the curing behavior by varying the elevation rate to 5, 10, 15, 20, and 30 °C/min within the range of 30–200 °C. Isothermal analysis was performed at the curing temperatures of 70 °C, 80 °C, and 90 °C.

The thermal stability and decomposition temperatures of the fully cured epoxy compound were measured via TGA using a Pyris 1 (Perkin Elmer, Waltham, MA, USA) thermal analyzer. The curing condition was 130 °C for 3 h. Samples weighing 5–10 mg were placed on a ceramic pan and heated in a nitrogen atmosphere to prevent oxidation. The heating process was performed at a constant rate of 10 °C/min from 30 °C to 800 °C.

A lap shear test was performed to measure the mechanical properties of the samples. The specimen consisted of an aluminum plate with an area of 25.4 × 10 mm^2^ coated with a 0.2 mm-thick layer of epoxy adhesive. The dimensions of the aluminum specimens used for the lap shear tests are shown in Figure S1. The test was conducted following ASTM D1002 using a universal testing machine model 5567 (Instron, Norwood, MA, USA) at a speed of 1.3 mm (0.05 in)/min. The specimens were cured at 130 °C for 3 h before testing in an oven. The lap shear strength was calculated as the mean value for five specimens for each configuration, with error bars representing one standard deviation.

## 3. Results and Discussion

### 3.1. Structural Analyses of TFPH and TFPM

**Figure 1:**
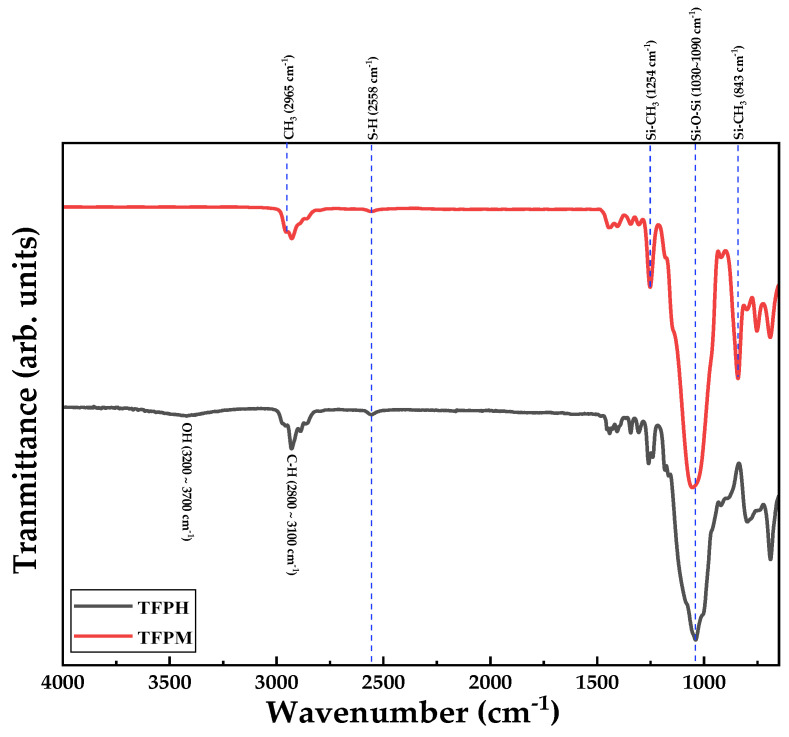
FTIR spectra of the synthesized TFPH and TFPM.

Figure 1 shows the FTIR spectra of TFPH and TFPM in the presence of various characteristic peaks, confirming the successful synthesis of the synthetic materials. The most prominent feature of TFPH and TFPM was the disappearance of the strong O–H stretching vibrations of the hydroxyl group near 3200 to 3700 cm^−1^ in TFPH and the appearance of sharp and intense peaks at 843 and 1257 cm^−1^ in TFPM, corresponding to Si–CH_3_ bending vibrations. These peaks were only detected with high intensity in TFPM. The FTIR spectra of TFPM also showed bands at 2965 cm^−1^ from the asymmetric and symmetric C–H stretching mode of CH_3_ groups. In both TFPH and TFPM, the mercapto S–H stretching mode of the mercapto groups showed a weak signal at 2556 cm^−1^ and the silsesquioxane showed strong peaks at 1030 to 1090 cm^−1^, indicating the presence of Si–O–Si bonds.

**Figure 2:**
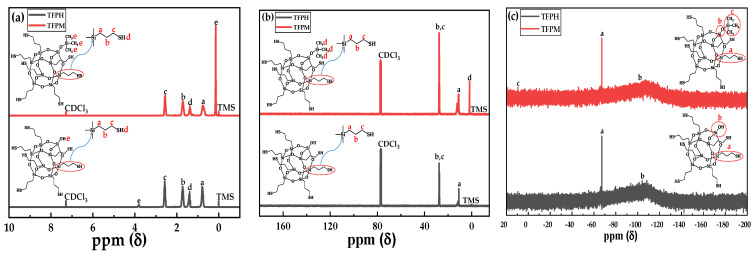
(**a**) ^1^H, (**b**) ^13^C, and (**c**) ^29^Si NMR spectra of TFPH and TFPM.

Figure 2a–c shows the ^1^H, ^13^C, and ^29^Si NMR spectra of TFPH and TFPM, respectively. In Figure 2a, the ^1^H NMR spectra of the synthesized TFPH and TFPM were similar to each other and showed peaks that confirmed the grafting of mercaptopropyl arms. The repeating unit of the mercaptopropyl chain has been assigned four signals: methylene protons a, b, and c and thiol protons d. On the basis of the ratio of the integral values of the peaks, the protons of TFPH and TFPM indicated that the ratio of H_a_:H_b_:H_c_:H_d_ at the mercaptopropyl chains was 1:1:1:0.5. The spectra of TFPH showed the presence of adsorbed water and hydrogen-bonded silanol groups (*δ* 3.8 ppm), whereas those of TFPM did not. In addition, the spectra of THPM exhibited a peak at *δ* 0.15 ppm attributable to the Si–CH_3_ group. The peak value of the chemical shifts [δ] was 7.3 ppm, which was calibrated to the CDCl_3_ solvent.

The ^13^C NMR spectra of TFPH and TFPM are depicted in Figure 2b. Similar to the ^1^H NMR spectra, the ^13^C NMR spectra of the synthesized TFPH and TFPM displayed peaks confirming the grafting of mercaptopropyl arms. The mercaptopropyl chain repeating unit was assigned two signals: methylene carbons a, and b, c in TFPH and TFPM. The spectrum of TFPM exhibited peaks at 1.39 ppm due to the Si–CH_3_ groups. In the ^13^C NMR chemical shift measurement, the 77 ppm triple line peaks were peaks shown because of the coupling of the carbon of solvent CDCl_3_ with deuterium (I = 1).

The ^29^Si NMR spectra of TFPH and TFPM in Figure 2c showed signals assigned to the T^3^ structure in a, indicating that the polyhedral oligomeric silsesquioxane (POSS) framework was preserved after poly-condensation. The signals at −64.13 to −68.75 ppm were assigned to T_7_-POSS. For both TFPH and TFPM, the peak at −102 to −110 ppm was observed and assigned to the Q unit in b. In addition, the spectrum of THPM showed a peak at −9.0 to −15 ppm assigned to the M unit in c.

The molecular weights of TFPH and TFPM were measured using GPC. As shown in Table 1, according to the GPC measurement with polystyrene as the standard, the average molecular weight of TFPH was 1032 g/mol, whereas the average molecular weight of TFPM was 1447 g/mol. The polydispersity indices (PDIs) of TFPH and TFPM were 1.059 and 1.404, respectively.

### 3.2. Comparative Analysis of the Thermal Characteristics of DPETMP, TFPH, and TFPM

**Figure 3:**
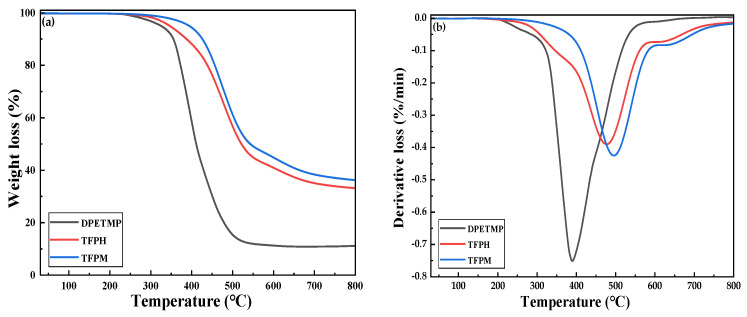
(**a**) TGA and (**b**) DTG curves of DPETMP, TFPH, and TFPM.

Figure 3a,b shows the decomposition curves of DPETMP, TFPH, and TFPM. The thermal safety factors of the composites were calculated from the thermogravimetric analysis (TGA) and are presented in Table 2. These factors include the following: the temperature of 5 wt% weight loss reduction (*T*_dec-5%_), the temperature of the maximum weight loss rate (*T*_max_), the thermal stability indices (A*, K*), and the integral procedural decomposition temperature (*IPDT*).

As shown in Table 2, the maximum decomposition temperatures of TFPH and TFPM were higher than those of DPETMP. At a 5% weight reduction, the temperatures were 334.49 °C for DPETMP, 352.32 °C for TFPH, and 402.35 °C for TFPM. The formation of silsesquioxane structures resulted in residual mass values of 33.95 and 36.54 wt% for TFPH and TFPM, respectively.

The integral procedural decomposition temperature (*IPDT*) is a quantitative indicator of thermal stability that Doyle suggested [30]. It is calculated as a ratio of areas and remains consistent and useful regardless of the number of steps in the decomposition process in TGA. The formula for obtaining the *IPDT* is shown in Equation (1).

*IPDT* (°C) = A^*^∙ K^*^ (*T_f_* –*T_i_*) + *T_i_* (1)

where,

A^*^ is the area ratio of total curve and total TGA thermogram ((*S*_1_ + *S*_2_)/(*S*_1_ + *S*_2_ + *S*_3_)),

K^*^ is the coefficient of A^*^, ((*S*_1_ + *S*_2_)/*S*_2_),

*T_i_* is the initial experimental temperature (30 °C), and

*T_f_* is the final experimental temperature (800 °C).

**Figure 4:**
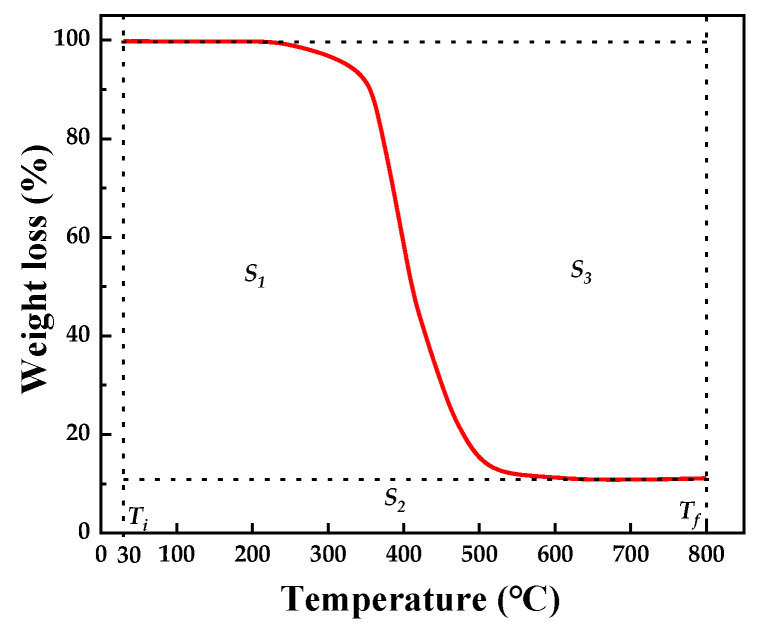
Schematic representation of *S*_1_, *S*_2_, and *S*_3_ for A^*^ and K^*^.

Figure 4 shows a schematic of determining the *IPDT*. *S*_2_ is the product of the residual amount and the temperature interval. *S*_1_ is the difference between the area under the decomposition curve and *S*_2_. *S*_3_ is the area that is not covered by the decomposition curve.

TFPH and TFPM had IPDT values of 678.49 °C and 702.03 °C, indicating excellent thermal stability and heat resistance. The high decomposition temperatures of the polysilsesquioxane backbone of TFPH and TFPM were due to the strong covalent Si–O–Si bonds, which resist thermal degradation. TFPM showed higher thermal stability than TFPH. Differences in the molecular weight may account for this. Polymers with a higher molecular weight tend to have stronger intermolecular forces, such as van der Waals forces [31], which make them more resistant to decomposition at high temperatures. Therefore, TFPM showed higher thermal stability than TFPH.

### 3.3. Comparison of Odor Intensities and VOC Emissions for DPETMP, TFPH, and TFPM

The odor intensities of DPETMP, TFPH, and TFPM were compared by five judges using the air dilution olfaction method [32]. The dilution factor for DPETMP was 25.42, while those for TFPH and TFPM were 8.04, indicating that TFPH and TFPM had weaker odors. The relevant dilution factors are shown in Tables S1–S3. TFPH and TFPM had higher molecular weights than DPETMP, which caused weaker thiol odors. The volatility and interaction of thiols with olfactory receptors in the nose affected their odors. Generally, there is a tendency for the vapor pressure to decrease with an increase in molecular weight [33]. Therefore, TFPH and TFPM had a lower volatility and weaker interaction with olfactory receptors than DPETMP. In addition, TFPM with the highest molecular weight had a lower probability of vaporization, which reduced their possibility of reaching the nose and binding to olfactory receptors [34]. This resulted in less intense odor perception.

The VOC factors of the compound are presented in Table 3. The factors include 5VOCs (an abbreviation for five volatile organic compounds considered harmful to human health and the environment: benzene, toluene, ethylbenzene, xylene, and styrene) and TVOC (an abbreviation for total volatile organic compounds, which is a measure of the total concentration of all VOCs and other organic compounds that can evaporate into the air and may or may not be harmful) [35,36]. Table 3 compares the volatile organic compound (VOC) emissions of the three types of thiol hardeners: the conventional commercial DPETMP and the newly synthesized TFPH and TFPM. DPETMP exceeded the Ecolabel and CertiPUR Label Requirements for TVOC emissions (minimum limit: 500 µg/m^3^), while TFPH and TFPM demonstrated low emissions of 138.25 and 118.21 µg/m^3^, respectively. As shown in Table 3, TFPH and TFPM had significantly lower VOC emissions than DPETMP. The higher molecular weights of TFPH and TFPM compared to DPETMP resulted in lower volatility and VOC emissions. TFPM had a higher molecular weight and lower VOC emissions than TFPH; therefore, TFPM had the lowest odor intensity among the three compounds.

### 3.4. Evaluating the Epoxy Cure Behavior of the 1-MI Curing Agent Alone and in Combination with Various Thiol-Based Hardeners

**Figure 5:**
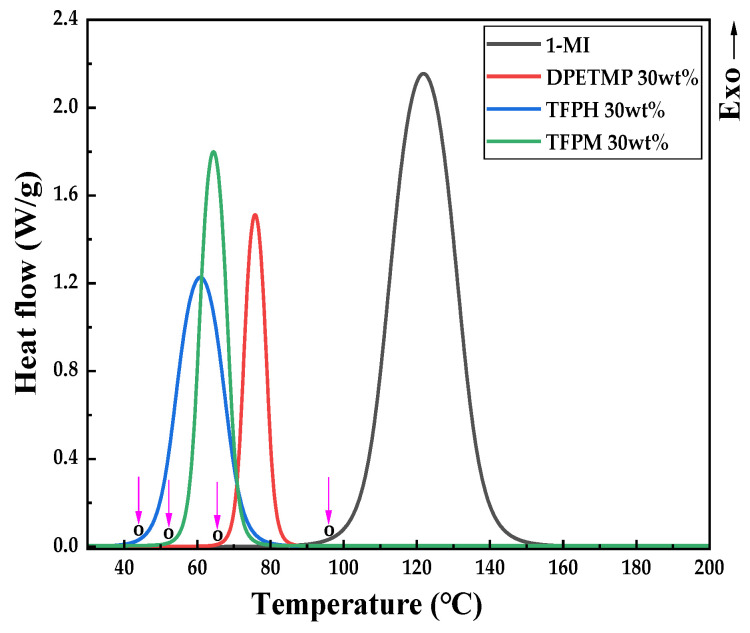
DSC traces at 5 °C/min for the curing of DGEBA with the 1-MI curing agent alone and in combination with various thiol-based hardeners (DPETMP, TFPH, and TFPM 30 wt%).

Thermosetting resins rely on the activation energy (Ea) to govern their cure reaction behaviors. The elevated temperature cure assay is often utilized because of its accuracy in determining the reaction rate within the cure initiation zone through a simple experimental setup to better understand such behaviors. In systems like epoxy, the exothermic peak shifts toward higher temperature regions as the heating rate of the DSC increases. For calculating the Ea of the curing reaction, Kissinger proposed a method that involves analyzing the movement of the reaction peak relative to the heating rate, which is expressed in Equation (2) [37].

(2) $$\frac{∆ln\left(\frac{q}{T_{p}^{2}}\right)}{∆\frac{1}{T_{p}}}=-\frac{E_{a}}{R}$$

where, *q* is the heating rate (°C/min), *T_p_* is the exothermic peak temperature (K), *E_a_* is the activation energy (Cal/mol), and *R* is the gas constant. In this study, thermal analysis was performed at different heating rates of 5, 10, 15, 20, and 30 °C/min to determine the *E_a_* and the total heat release (Δ*H*_0_). Experiments were conducted with various DPETMP weight percentages to set the amount of the thiol-based hardener, and the data for this are shown in Figure S2 and Table S4. In this study, the addition of the thiol hardener was set to 30 wt% based on Figure S2 and Table S4. The onset of exothermicity was determined based on the inflection point of the exothermic curve, which was denoted as point O in Figure 5 and represented the reaction onset temperature (*T*_onset_). The results showed that 1-MI had the highest curing temperature with a *T*_onset_ of about 98 °C. DPETMP had a *T*_onset_ of 67 °C, whereas TFPH and TFPM had lower *T*_onset_ values of 45 °C and 53 °C, respectively. Table 4 shows the *T*_onset_, exothermic peak temperature (*T_p_*), and total calorific value (Δ*H*_0_) at each heating rate as can be measured in Figure 5. The heat flow obtained from the area under the DSC curve was determined as the average of three values obtained at the curing rates of 5, 10, 15, 20, and 30 °C/min. We found that the number of reactive groups in the thiol hardeners affected the cure temperature of the epoxy resins. TFPH and TFPM with 7 thiol-reactive groups had lower curing temperatures than DPETMP with 6 thiol-reactive groups. The hydroxyl groups in TFPH acted as nucleophiles, which attacked the electrophile epoxy groups to promote ring-opening polymerization. Among all the thiol hardeners, TFPH exhibited the lowest initiation temperature on DSC because of the presence of hydroxyl groups that facilitated ring-opening polymerization [38].

**Figure 6:**
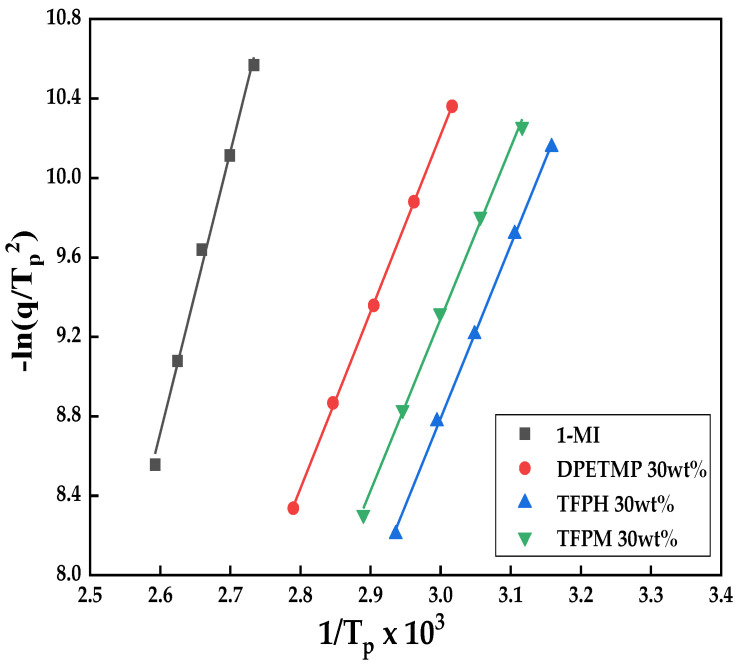
Plots of −ln(q/T_p_^2^) vs. (1/T_p_) × 10^3^ based on the Kissinger equation for the curing of DGEBA with the 1-MI curing agent alone and in combination with various thiol-based hardeners (DPETMP, TFPH, and TFPM 30 wt%).

*E*_a_ is plotted in Figure 6 as −ln(*q*/*T*_p_^2^) vs. (1/*T*_p_) × 10^3^. Each point shows a linear change, and *E*_a_ can be obtained from the slope of this straight line. They are calculated and summarized in Table 5. *E*_a_ was obtained from the slope of each value. *E*_a_ was dependent on the types of hardeners used. For 1-MI alone, the highest value of *E*_a_ was 42.12 kcal/mol, followed by that of DPETMP at 29.12 kcal/mol and those of TFPH and TFPM at 23.20 and 25.24 kcal/mol, respectively. These results suggest that *E*_a_ decreased following the order 1-MI > DPETMP > TFPM > TFPH. The number of reactive thiol groups in the hardener significantly influenced the *E*_a_ of the thiol–epoxy reaction. TFPH and TFPM with 7 thiol-reactive groups had lower activation energies compared to DPEMP with 6 thiol-reactive groups. This was consistent with the lower cure initiation temperatures observed for TFPH and TFPM. Moreover, TFPH had the lowest *E*_a_ value among the hardeners, which we ascribed to the catalytic effect of the hydroxyl groups in its structure. The hydroxyl groups could form hydrogen bonds with the epoxy resin, thereby increasing the reactivity and facilitating the thiol–epoxy reaction [39]. However, when excessive amounts of DPETMP, TFPH, and TFPM were added to the epoxy, some of the thiol groups underwent a thiol exchange reaction with the thioether linkages formed by the thiol-epoxy reaction, leading to the formation of dimers and trimers [40]. These newly formed dimers and trimers had lower reactivity toward epoxy groups, which hindered the radical initiation of the thiol-epoxy reaction [41]. Therefore, as the thiol hardener content increased, the curing temperature and *E*_a_ required for the reaction to occur also increased [42]. This is evident in Figure S3 and Table S5, which show the kinetic coefficients and calculated activation energies obtained from the Kissinger model, respectively, analyzed as a function of the thiol hardener content (wt%). *E*_a_ increased from 40 wt%, indicating that the reaction became more difficult to initiate at higher thiol hardener contents.

Isothermal DSC analysis can determine the time for the curing reaction to be completed at a specific temperature. Investigating the change in the curing degree with time at a specific temperature is critical to establishing optimal curing conditions for processing thermoset resins. Generally, isothermal methods are simpler and more reliable than kinetic ones for analyzing data and deriving reaction rate parameters. When measuring the curing reaction rate using DSC, the amount of epoxy consumed during the curing reaction is assumed to be proportional to the calorific value. Therefore, the calorific value of the curing reaction (*H*) is proportional to the reaction conversion rate (*α*), which is expressed in Equation (3). The reaction rate, i.e., the rate at which α cures (*dα/dt*), is expressed in Equation (4). *α* can also be expressed as the degree of curing [43,44].

(3) $$\alpha =\frac{∆H_{t}}{∆H_{0}}$$

(4) $$\frac{d\alpha }{dt}=\frac{{\left(\frac{dH}{dt}\right)}_{T}}{∆H_{0}}$$

**Figure 7:**
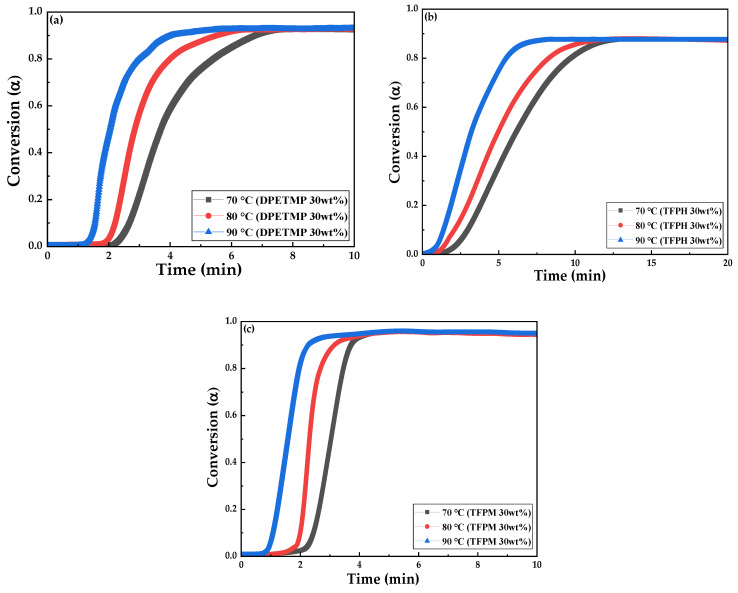
Conversion behaviors at various curing temperatures (70 °C, 80 °C, and 90 °C) along with the comparison of curing times with thiol-based hardeners: (**a**) DPETMP, (**b**) TFPH, and (**c**) TFPM.

Figure 7a–c shows the change in the degree of the curing reaction of DPETMP, TFPH, and TFPM at 70 °C, 80 °C, and 90 °C with time. The area of the total calorific value was integrated over time to analyze the change in the curing degree over time. This curing reaction followed an autocatalytic reaction model, as the curing curve showed an S-shaped curve where the reaction rate was slow at the beginning of curing, rapidly accelerated, and gently slowed down at the end [45].

**Figure 8:**
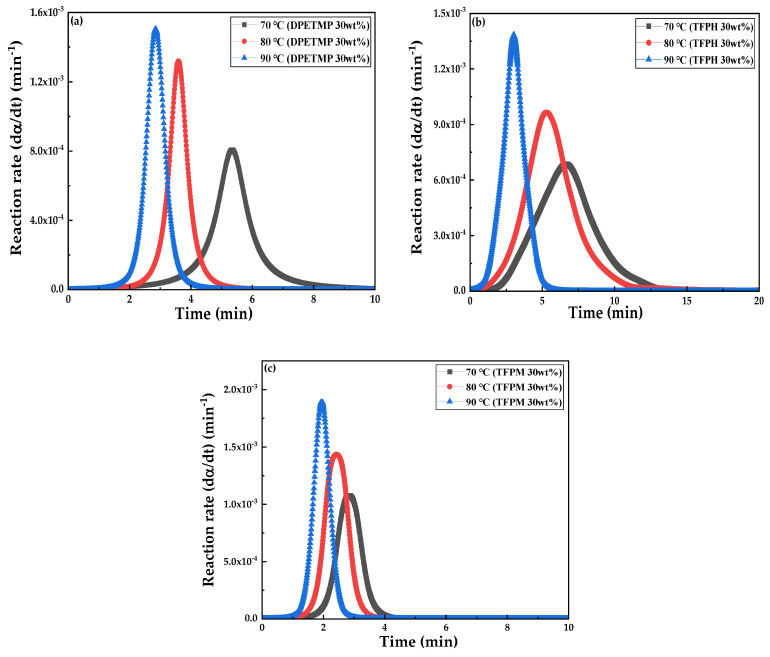
Comparison of the curing rates with thiol-based hardeners. (**a**) DPETMP, (**b**) TFPH, and (**c**) TFPM at various curing temperatures: 70 °C, 80 °C, and 90 °C.

The reaction start time was faster and the degree of curing increased rapidly as the temperature increased, indicating that the higher the curing temperature, the faster the curing reaction was completed. The steepest slopes of the curves indicated the fastest crosslinking reaction rates at 90 °C curing conditions and occurred between 1.6 and 4.3 min for DPETMP, 0.98 and 5.05 min for TFPH, and 1.16 and 2.76 min for TFPM. The crosslinking reaction rates of the three thiol-based hardeners (DPETMP, TFPH, and TFPM) at different curing temperatures (70 °C, 80 °C, and 90 °C) are plotted in Figure 8 as a function of time to compare their curing kinetics. The maximum rate and time of the curing reaction for each hardener at each temperature are summarized in Table 6.

We observed that TFPH and TFPM with 7 thiol-reactive groups initiated the reaction faster than DPETMP with 6 thiol-reactive groups. However, despite its faster initial reaction, TFPH had a slower final curing time and lower conversion than those of DPETMP and TFPM. We attributed this to the effect of the hydroxyl groups in TFPH, which facilitated the ring-opening reaction at the beginning of the curing process by forming hydrogen bonds with the epoxy resin but later competed with the thiol groups for the epoxy ring-opening reaction, thus reducing the reaction rate and efficiency [46].

### 3.5. Thermal Stability Analysis of the Epoxy Composite with the 1-MI Curing Agent Alone and in Combination with Different Thiol-Based Hardeners

**Figure 9:**
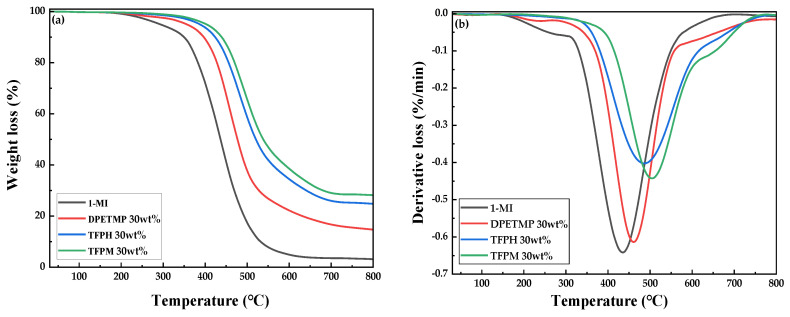
Thermograms of the epoxy composite with the 1-MI hardener alone and in combination with various thiol-based hardeners from (**a**) TGA and (**b**) DTG.

Figure 9a,b shows the TGA and derivative thermogravimetry (DTG) curves of the epoxy composite cured with the 1-MI curing agent alone and in combination with different thiol-based hardeners, respectively. The thermal safety factors of the composites, such as the temperature of 5 wt% weight loss reduction (*T*_dec-5%_), the temperature of the maximum weight loss rate (*T*_max_), the thermal stability indices (A^*^, K^*^), and the *IPDT*, were calculated from the thermal analysis and are presented in Table 7. TFPH and TFPM had strong Si–O–Si covalent bonds in their polysilsesquioxane backbones, which limited heat transfer from the polymer surface to the internal structure and prevented localized thermal degradation.

As shown in Figure 3a,b and Table 2, TFPM had the highest thermal stability among the compounds tested. TFPM, which has a relatively high molecular weight, can resist degradation at high temperatures better than the other compounds due to its strong intermolecular attraction.

### 3.6. Analysis of the Mechanical Properties of the Epoxy Compound with the 1-MI Hardener Alone and in Combination with Different Thiol-Based Hardeners

**Figure 10:**
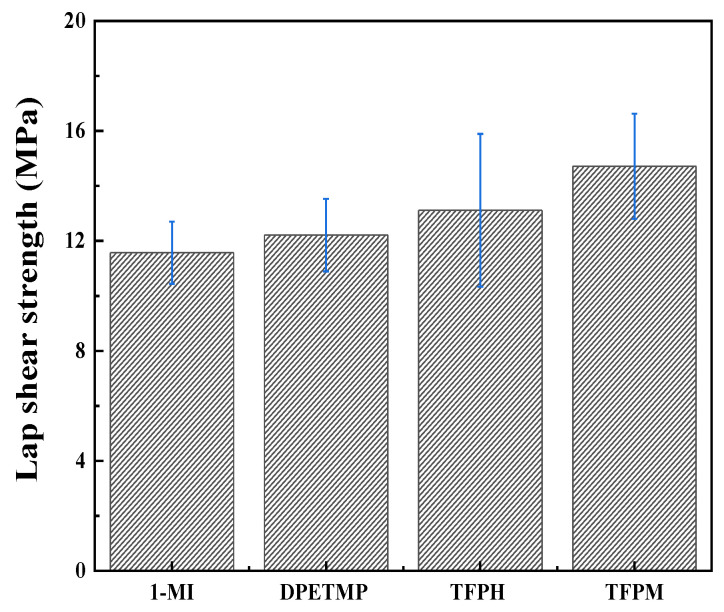
Lap shear strength with the 1-MI curing agent alone and in combination with different thiol-based hardeners (DPETMP, TFPH, and TFPM 30 wt%).

We measured the lap shear strength of samples cured with either 1-MI alone or 1-MI mixed with 30 wt% of DPETMP, TFPH, or TFPM to evaluate the impact of thiol-based hardeners on the mechanical strength of the epoxy compound. Figure 10 compares the lap shear strengths of the epoxy cured with 1-MI alone with the addition of various thiol-based hardeners. The lap shear strength increased following the order TFPM > TFPH > DPETMP > 1-MI. The epoxy compound cured with TFPM showed the highest lap shear strength of 14.71 MPa, a 20% improvement over that of the epoxy compound cured with DPETMP (12.21 MPa).

These results are similar to those reported in previous literature [47,48]. The introduction of the POSS structure improved the adhesive performance of epoxy, which was also confirmed in this study. The molecular weight of a polymer influences its thermal, mechanical, and rheological properties. Furthermore, higher molecular weight polymers also influenced the adhesive’s wetting, adhesion, and cohesion properties [49,50]. Therefore, TFPM, with a relatively high molecular weight compared to the other compounds, showed the best adhesion performance. However, relatively high error bars of TFPH and TFPM measurements and the complex interactions of various factors influencing adhesion performance suggest further study.

## 4. Conclusions

In this study, a novel thiol-based hardener with low odor, low VOC emissions, and fast curing at low temperatures was successfully developed through synthesis using the acid-catalyzed sol–gel method. We found that the number of reactive thiols and their molecular weights are important factors. TFPH and THPM have 7 reactive thiol groups and lower cure temperatures than commercial hardeners with 6 reactive thiol groups. The hydroxyl groups in TFPH promote ring-opening polymerization, resulting in the fastest cure initiation temperature and reaction time. However, due to competition between hydroxyl and thiol groups during curing, the final cure time and conversion rate were lower than those of other hardeners. On the other hand, TFPM, with the highest molecular weight, showed the lowest VOC emissions due to the decrease in volatility due to the decrease in vapor pressure, and the thermal stability and adhesion performance was found to be higher than those of other curing agents due to the stronger intermolecular forces. Moreover, TFPM had a higher TGA at a 5% weight loss temperature (>50 °C) and lap shear strength (20%) than those of the epoxy compounds with the commercial hardener.

The newly developed organic–inorganic hybrid epoxy resin offers significant benefits. First, it reduces the emission of volatile organic compounds (VOCs) and odors without compromising its performance, thereby enhancing the cleanliness and safety of the working environment. Second, the resin exhibits higher thermal stability than conventional epoxy adhesives, enabling its application in more demanding conditions, such as high temperatures or chemical exposure.

Consequently, the newly developed thiol-based hardeners could be a promising alternative as an epoxy hardener by solving the problems of commercially available thiol-based hardeners and improving their performance.

## Figures and Tables

**Table 1:** Molecular weights and polydispersity indices determined using GPC in THF based on a polystyrene calibration.

	M_n_ (g/mol)	M_w_ (g/mol)	M_z_ (g/mol)	PDI (PolyDispersity Index) [Mw/Mn]
**TFPH**	982	1032	1138	1.059
**TFPM**	1030	1447	2250	1.404

**Table 2:** Thermal data obtained from the TGA thermograms of DPETMP, TFPH, and TFPM.

	*T_dec-5%_* (°C)	*T_max_* (°C)	A*· K*	*IPDT* (°C)	Residual Mass (%)
**DPETMP**	334.49	388.49	0.52	514.45	11.01
**TFPH**	352.32	477.84	0.61	678.59	33.95
**TFPM**	402.35	495.05	0.65	702.03	36.54

**Table 3:** VOC emission comparison among DPETMP, TFPM, and TFPH.

[µg/m^3^]	DPETMP	TFPH	TFPM
**TVOC**	1060	138.25	118.25
**5VOCs**	4.1	4.7	4.2
**Toluene**	0.41	1.1	0.8
**Benzene**	3.64	3.6	3.3
**Ethylbenzene**	0	0	0
**m,p-Xylene**	0	0	0
**Styrene**	0	0	0

**Table 4:** Initial temperature (*T*_Onset_), peak temperature (*T*_p_), and heat flow for 1-MI, DPETMP, TFPH, and TFPM 30 wt%.

Sample	T (°C)	Heating Rate (°C/min)					ΔH (kJ/mol)
		5	10	15	20	30	
**1-MI**	T_onset_	98.10	104.21	110.28	114.29	122.21	412.63
	T_Peak_	123.64	127.68	130.39	134.65	138.19	
	T_endset_	150.55	154.53	157.89	161.22	165.16	
**DPETMP 30 wt%**	T_Onset_	63.02	70.08	77.29	84.56	92.07	229.75
	T_Peak_	74.42	77.61	81.55	86.31	91.39	
	T_endset_	83.63	92.36	99.47	106.12	112.04	
**TFPH 30 wt%**	T_Onset_	43.79	48.35	58.71	62.29	69.24	281.82
	T_Peak_	61.27	63.29	68.11	71.41	73.17	
	T_endset_	79.32	88.52	98.19	104.66	118.02	
**TFPM 30 wt%**	T_Onset_	52.73	64.14	70.71	76.89	83.24	262.91
	T_Peak_	64.21	69.21	72.11	74.39	75.79	
	T_endset_	72.84	82.52	88.84	92.15	98.48	

**Table 5:** Kinetic factors obtained from the Kissinger model and calculated activation energies.

Sample	Kinetic Factor	Heating Rate (°C/min)					E_a_ (kcal/mol)
		5	10	15	20	30	
**1-MI**	(1/T_P_) × 10^3^	2.733	2.699	2.659	2.624	2.592	42.12
	ln (q/T_p_^2^)	10.56	10.11	9.63	9.07	8.55	
**DPETMP 30 wt%**	(1/T_P_) × 10^3^	3.016	2.961	2.904	2.846	2.790	29.12
	ln (q/T_p_^2^)	10.36	9.88	9.35	8.86	8.33	
**TFPH 30 wt%**	(1/T_P_) × 10^3^	3.158	3.105	3.048	2.994	2.935	23.20
	ln (q/T_p_^2^)	10.15	9.71	9.92	8.87	8.20	
**TFPM 30 wt%**	(1/T_P_) × 10^3^	3.116	3.056	2.999	2.945	2.889	25.24
	ln (q/T_p_^2^)	10.25	9.80	9.31	8.83	8.30	

**Table 6:** Maximum rate and time for the curing reaction with 30 wt% addition of DPETMP, TFPH, and TFPM at different temperatures of 70 °C, 80 °C, and 90 °C.

Temperature (°C)	DPETMP 30 wt%		TFPH 30 wt%		TFPM 30 wt%	
	Time (min)	Maximum Rate (min^−1^)	Time (min)	Maximum Rate (min^−1^)	Time (min)	Maximum Rate (min^−1^)
**70**	5.32	8.27 × 10^−4^	6.63	7.02 × 10^−4^	2.81	1.43 × 10^−3^
**80**	3.57	1.34 × 10^−3^	5.25	9.25 × 10^−4^	2.40	1.46 × 10^−3^
**90**	2.84	1.53 × 10^−3^	3.01	1.38 × 10^−3^	1.92	1.95 × 10^−3^

**Table 7:** Thermal stability of the epoxy composite with 1-MI alone and in combination with various thiol-based hardeners (DPETMP, TFPH, and TFPM 30 wt%).

	*T_dec-5%_* (°C)	*T_max_* (°C)	A^*^· K^*^	*IPDT* (°C)	Residual Mass (%)
**1-MI**	293.86	433.25	0.53	580.72	3.35
**DPETMP**	349.16	461.17	0.61	613.76	14.35
**TFPH**	386.09	485.22	0.72	678.35	24.83
**TFPM**	408.45	504.53	0.77	728.01	28.58

## Data Availability

The data are available upon reasonable request from the corresponding author.

## Supplementary Materials

The following supporting information can be downloaded at: https://www.mdpi.com/article/10.3390/polym15132947/s1, Figure S1: Dimensions of the lap shear specimens used in this study.; Table S1: Dilution factor of DPETMP.; Table S2: Dilution factor of TFPH.; Table S3: Dilution factor of TFPM.; Figure S2: DSC traces at 5 °C/min for the cure of DGEBA with the 1-MI curing agent alone and in combination with various weight percentages of DPETMP.; Table S4: Initial temperature (*T*_Onset_), peak temperature (*T*_p_), and heat flow for each weight percentage of DPETMP.; Figure S3: Plots of −ln(*q*/*T*_p_*^2^*) vs. (1/*T*_p_) × 10^3^ based on the Kissinger equation for the curing of DGEBA with the 1-MI curing agent alone and in combination with various weight percentages of DPETMP.; Table S5: Kinetic coefficients and calculated activation energies obtained from the Kissinger model as a function of the weight percentage content of DPETMP.
