# Supplementary material for: Effect of Synthetic Low-Odor Thiol-Based Hardeners Containing Hydroxyl and Methyl Groups on the Curing Behavior, Thermal, and Mechanical Properties of Epoxy Resins
Source: Polymers (Basel). 2023 Jul 4;15(13):2947. doi: 10.3390/polym15132947 (PMC10346956; doi:10.3390/polym15132947)
Supplement: Supplementary file 1 [file polymers-15-02947-s001.zip › Polymers 2476771-Supplementary.pdf]

Supporting information

Effect of synthetic low-odor thiol-based hardeners containing hydroxyl and methyl groups on the curing behavior, thermal, and mechanical properties of epoxy resins

Young-Hun Kim[1,2], Jeong Ju Baek[1], Ki Cheol Chang[1], Baek Soo Park[1], Won-Gun Koh[2,*], Gyojic Shin[1,*]

[1]Green and Sustainable Materials R&D Department Research Institute of Clean Manufacturing System, Korea Institute of Industrials Technology (KITECH), Yangdaegiro-gil 89, Ipjang-myeon, Cheonan-si 31056, South Korea

[2]Department of Chemical and Biomolecular Engineering, Yonsei University, Yonsei-ro 50, Seodaemun-gu, Seoul 03722, South Korea

[*]Corresponding author: wongun@yonsei.ac.kr, gyshin@kitech.re.kr

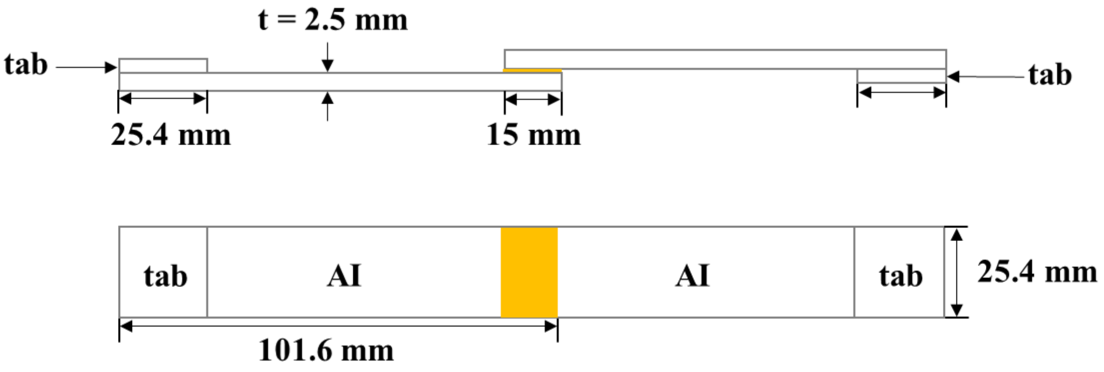

Figure S1. Dimensions of the lap shear specimens used in this study

Table S1. Dilution factor of DPETMP.

Judge	Calculation	Remarks	Dilution Factor
**A**	A = √(10) = 5.447	Least (Except)	
**B**	B = 100	→	
**C**	C= √(10) = 5.447	→	$\sqrt[3]{(5.447 * 100 * 30)} = 25.42$
**D**	D = 30	→	
**E**	E = 300	Maximum (Except)	

Table S2. Dilution factor of TFPH.

Judge	Calculation	Remarks	Dilution Factor
**A**	A = √(3) = 1.732	Least (Except)	
**B**	B = 100	Maximum (Except)	
**C**	C= √(3) = 1.732	→	$\sqrt[3]{(1.732 * 30 * 10)} = 8.04$
**D**	D = 10	→	
**E**	E = 30	→	

Table S3. Dilution factor of TFPM.

Judge	Calculation	Remarks	Dilution Factor
**A**	A= 100	Maximum (Except)	
**B**	B = 10	→	
**C**	C = $\sqrt{(3)}$ = 1.732	→	$\sqrt[3]{(1.732 * 30 * 10)} = 8.04$
**D**	D = $\sqrt{(3)}$ = 1.732	Least (Except)	
**E**	E = 30	→	

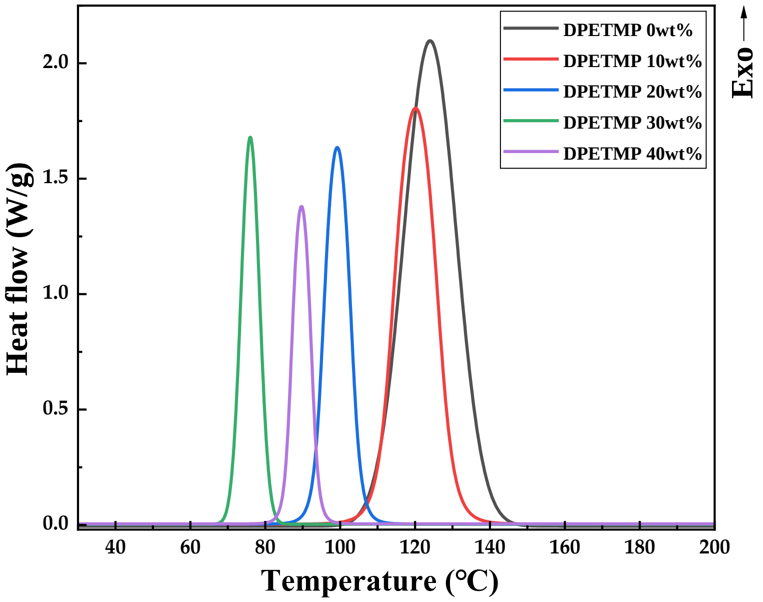

Figure S2. DSC traces at 5°C/min for the cure of DGEBA with the 1-MI curing agent alone and in combination with various weight percentages of DPETMP.

Table S4. Initial temperature (T_{Onset}), peak temperature (T_P), and heat flow for each weight percentage of DPETMP.

Sample	T (℃)	Heating rate (℃/min)					ΔH (kJ/mol)
		5	10	15	20	30	
DPETMP 0wt%	T_{Onset}	98.10	104.21	110.28	114.29	122.21	412.63
	T_{Peak}	123.64	127.68	130.39	134.65	138.19	
	T_{endset}	150.55	154.53	157.89	161.22	165.16	
DPETMP 10wt%	T_{Onset}	95.28	100.21	106.17	112.98	118.43	353.08
	T_{Peak}	120.14	124.88	129.74	134.25	138.63	
	T_{endset}	143.60	149.33	154.18	160.66	166.91	
DPETMP 20wt%	T_{Onset}	82.26	87.11	94.16	101.23	109.76	263.51
	T_{Peak}	99.08	103.02	108.59	112.44	117.09	
	T_{endset}	116.02	122.49	128.12	136.37	143.81	
DPETMP 30wt%	T_{Onset}	63.02	70.08	77.29	84.56	92.07	229.75
	T_{Peak}	76.39	77.61	81.55	86.31	91.39	
	T_{endset}	83.63	92.36	99.47	106.12	112.04	
DPETMP 40wt%	T_{Onset}	79.92	87.88	95.11	102.52	108.17	185.15
	T_{Peak}	89.52	94.42	98.45	102.01	107.38	
	T_{endset}	102.59	106.99	113.68	118.13	125.69	

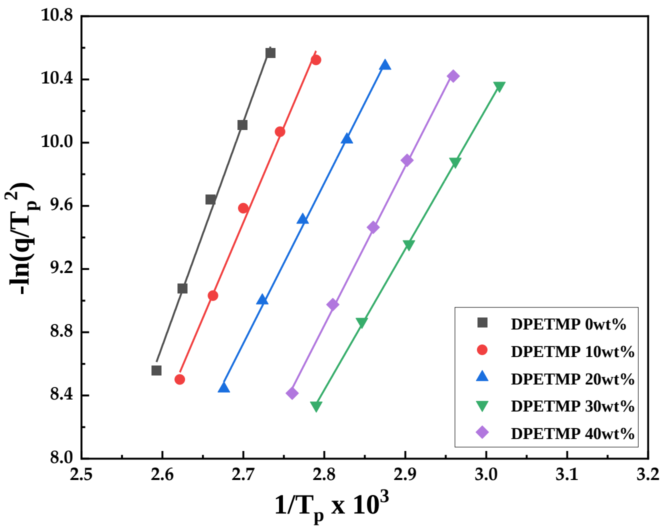

Figure S3. Plots of $-\ln(q/T_P^2)$ vs. $(1/T_P) \times 10^3$ by the Kissinger equation for the curing of DGEBA with the 1-MI curing agent alone and in combination with various weight percentages of DPETMP.

Table S5. Kinetic coefficients and calculated activation energies obtained from the Kissinger model as a function of the weight percentage content of DPETMP.

Sample	Kinetic factor	Heating rate (℃/min)					E_a (kcal/mol)
		5	10	15	20	30	
**DPETMP 0wt%**	$(1/T_P) \times 10^3$	2.733	2.699	2.659	2.624	2.592	42.12
	$\ln (q/T_P^2)$	10.56	10.11	9.63	9.07	8.55	
**DPETMP10wt%**	$(1/T_P) \times 10^3$	2.789	2.745	2.712	2.662	2.621	40.15
	$\ln (q/T_P^2)$	10.52	10.06	9.58	9.03	8.53	
**DPETMP 20wt%**	$(1/T_P) \times 10^3$	2.875	2.827	2.773	2.723	2.675	36.36
	$\ln (q/T_P^2)$	10.48	10.01	9.51	9.18	8.44	
**DPETMP 30wt%**	$(1/T_P) \times 10^3$	3.016	2.961	2.904	2.846	2.791	29.12
	$\ln (q/T_P^2)$	10.36	9.88	9.35	8.86	8.33	
**DPETMP40wt%**	$(1/T_P) \times 10^3$	2.959	2.902	2.862	2.811	2.765	31.29
	$\ln (q/T_P^2)$	10.42	9.83	9.46	8.97	8.41	
